# Marked variation in the bacterial microbiota of commercially produced Greek kefir products revealed by full-length 16S rRNA gene sequencing

**DOI:** 10.3389/fmicb.2026.1951948

**Published:** 2026-10-07

**Authors:** Despina Vougiouklaki, Panagiotis Lamprou, Nikos Mouhtouris, Marialena Fonazaki, Anthimia Batrinou, Spyros J. Konteles, Monika Polanska, Jan F. M. Van Impe, Efstathia Tsakali, Dimitra Houhoula

**Affiliations:** 1 Department of Food Science and Technology, Faculty of Food Sciences, University of West Attica, Egaleo, Greece; 2 Department of Chemical Engineering, BioTeC—Chemical and Biochemical Process Technology and Control, KU Leuven, Gent, Belgium

**Keywords:** 16S rRNA, Greek kefir, lactic acid bacteria, microbial diversity, Oxford Nanopore sequencing

## Abstract

Kefir is a fermented dairy beverage characterized by a complex microbial community that contributes to its technological and sensory properties and has been associated in previous studies with potential health-related effects. While the microbiota of kefir grains has been extensively studied, information regarding the bacterial communities of commercially produced Greek kefir remains limited. The aim of the present study was to characterize the bacterial community composition of commercially produced kefir products collected from different regions of Greece using full-length 16S rRNA gene sequencing and to assess variation among the analyzed commercial product groups. A total of 32 kefir samples were collected from eight regions of Greece: Koufalia, Kilkis, Drama, Thrace, Katerini, Larissa, Volos, and Attica. Genomic DNA was extracted and subjected to Oxford Nanopore full-length 16S rRNA gene sequencing. Taxonomic profiling was performed following quality filtering and bioinformatic analysis. The bacterial communities were predominantly composed of members of the phylum Bacillota, representing more than 92% of the total bacterial relative abundance in all samples. At the species level, *Streptococcus thermophilus* (45.89%) and *Lactococcus cremoris* (37.72%) were identified as the dominant shared taxa. Bray–Curtis dissimilarities and PERMANOVA supported differentiation among the commercial product groups (pseudo-*F* = 5.630, *R*^2^ = 0.622, and permutation *p* = 0.001). Kefir samples from Koufalia were strongly dominated by *L. cremoris* (88.59%), whereas samples from Attica, Thrace, and Kilkis showed a predominance of *S. thermophilus*. In contrast, Katerini showed a compositionally heterogeneous bacterial profile with comparatively low dominance of the two core taxa. It also presented relatively higher abundances of *Leuconostoc* spp., *Lacticaseibacillus rhamnosus, Lactobacillus delbrueckii*, and *Bifidobacterium animalis*. These findings demonstrate that commercially produced Greek kefir products share dominant bacterial taxa while exhibiting marked variation among commercial product groups. The observed variation among commercial product groups may be associated with product- and manufacturer-related factors, although the relative contribution of these factors cannot be determined from the present dataset.

## 1 Introduction

Kefir is a traditional fermented dairy beverage widely recognized for its unique sensory characteristics and potential health-promoting properties. It is produced through the fermentation of milk by a complex symbiotic consortium of microorganisms, mainly consisting of lactic acid bacteria (LAB), yeasts, and occasionally acetic acid bacteria (Guzel-Seydim et al., 2005; Simova et al., 2002; Kazou et al., 2021; Azizi et al., 2021). The microbial composition of kefir may vary between kefir grains, the final fermented beverage, and products manufactured using defined starter cultures, reflecting interactions among LAB and yeasts during fermentation (Simova et al., 2002; Beshkova et al., 2002; Sarkar, 2008). Studies of kefir products from different countries have also reported variable bacterial and yeast profiles, further supporting the dynamic nature of kefir microbiota (Sefidgar et al., 2014; Altay et al., 2013).

The microbial composition of kefir may be influenced by factors such as milk type, fermentation conditions, starter culture composition, and production practices; apparent geographical differences may also reflect differences in starter culture formulations, production practices, and local manufacturing conditions (Kalamaki and Angelidis, 2020; Kazou et al., 2021; Arslan, 2015). In addition, the type of milk and the culture used during kefir manufacture may affect the physicochemical, rheological, and sensory characteristics of the final product (Wszolek et al., 2001; Öner et al., 2010; Tratnik et al., 2006; de Sainz et al., 2020; Barukčić et al., 2017).

Previous studies investigating Greek kefir microbiota demonstrated considerable variability between artisanal kefir grains and industrial kefir beverages. Kalamaki and Angelidis (2020) reported that Greek kefir grains were predominantly dominated by *Lactobacillus kefiranofaciens*, followed by *Lactobacillus kefiri* and *Lactobacillus parakefiri*, whereas Kazou et al. (2021) observed that industrial Greek kefir beverages were mainly characterized by the predominance of *Streptococcus thermophilus, Lactobacillus delbrueckii* subsp. *bulgaricus*, and *Lacticaseibacillus rhamnosus*.

In recent years, kefir has attracted increasing scientific interest due to its potential biological activities, including antimicrobial, antioxidant, anti-inflammatory, immunomodulatory, and probiotic effects, which are mainly associated with its diverse microbial ecosystem and fermentation-derived metabolites (Otles and Cagindi, 2003; Ahmed et al., 2013; Guzel-Seydim et al., 2011; Azizi et al., 2021). More broadly, probiotics have been associated with potential health-related effects, including modulation of gut microbiota composition, enhancement of intestinal barrier function, and immune-related effects (Kechagia et al., 2013). Furthermore, recent systematic reviews of randomized controlled trials suggest that kefir consumption may exert beneficial effects on human health, although further clinical evidence is still required to fully elucidate its mechanisms of action and therapeutic applications (Kairey et al., 2023). However, the detection of bacterial taxa by DNA-based 16S rRNA gene sequencing does not itself demonstrate microbial viability, probiotic functionality, or health-related activity, which require complementary culture-based, functional, or metabolomic approaches.

Previous studies have suggested that differences in kefir microbial consortia may be associated with differences in fermentation-derived metabolites and biological effects; however, such functional properties cannot be inferred from taxonomic profiling alone. In a mouse model of diet-induced obesity, Bourrie et al. (2021) demonstrated that kefir products with different microbial compositions produced distinct effects on cholesterol and lipid metabolism, highlighting the importance of microbial interactions during fermentation. In addition, regular kefir consumption has been associated with changes in selected gut microbiota-related outcomes in human subjects (Bellikci-Koyu et al., 2019). Kefir-derived mixtures of LAB and yeasts have also demonstrated retained viability and *in vitro* probiotic-related properties following freeze-drying, further highlighting the functional relevance of kefir-associated microbial consortia (Bolla et al., 2011). Kefir-associated microflora may also be exploited for the production of functional microbial biomass and bioactive compounds, further demonstrating the technological potential of the kefir ecosystem (Paraskevopoulou et al., 2003).

The composition of kefir starter cultures may also influence the technological, rheological, and sensory characteristics of the final product. de Sainz et al. (2020) showed that kefir produced using different kefir grains exhibited differences in physicochemical, rheological, and sensory properties. Similarly, Barukčić et al. (2017) reported differences in chemical, rheological, and sensory properties between kefir produced with traditional kefir grains and kefir produced with a commercial starter culture. Storage may also influence the microbiological and chemical characteristics of kefir products, further contributing to variation in the final product profile (Grønnevik et al., 2011). These findings support the importance of detailed microbiome characterization of commercially produced kefir products.

Recent investigations of retail kefir products have highlighted substantial variation in microbial composition among commercially available products. Using full-length amplicon Nanopore sequencing, Maughan et al. (2025) characterized bacterial and fungal communities in retail kefirs in Ireland and reported considerable variation among products. Similarly, Metras et al. (2021) demonstrated that commercial kefir products may differ in bacterial composition and that product labels do not always fully reflect the taxa detected by sequencing-based analysis. These findings underline the importance of microbiome-based characterization of commercial kefir products and support comparative studies examining variation among products associated with different manufacturing settings.

The aim of the present study was to characterize the bacterial community composition of commercially produced Greek kefir products collected from different regions of Greece using full-length 16S rRNA gene sequencing. In addition to describing the dominant bacterial taxa, the study compared the analyzed commercial product groups to assess variation in bacterial community composition. The use of full-length 16S rRNA gene sequencing enabled improved taxonomic resolution, particularly at the species level, compared with short-read amplicon approaches. This study therefore provides a comparative overview of the bacterial microbiota of commercially produced Greek kefir and highlights marked variation among commercial product groups, while product- and manufacturer-related factors are considered possible contributors rather than demonstrated causes of the observed differences.

## 2 Materials and methods

### 2.1 Sample collection

A total of 32 commercially produced kefir samples were collected from eight regions of Greece: Koufalia, Kilkis, Drama, Thrace, Katerini, Larissa, Volos, and Attica, over a 3-month sampling period. Four independent production batches of the same commercial kefir product were collected from each sampling location, resulting in a total of 32 samples. Each sampling group therefore represented one commercial product from a single manufacturer, analyzed across four separate production batches. Manufacturer identities were anonymized. Available product metadata, including milk source, declared starter culture or microorganisms, storage conditions, product formulation, and other label information, are provided in Supplementary Table S1. All products originated from industrial-scale production facilities and were purchased from local retail markets. Samples were transported to the laboratory under refrigerated conditions at 4 °C and processed immediately upon arrival. Laboratory analyses were performed in duplicate to assess analytical reproducibility. These analytical duplicates were not treated as independent biological observations and were not included as separate samples in downstream statistical analyses. All diversity and inferential statistical analyses were conducted using the 32 independent production batches, corresponding to four independent batches per commercial product group. The geographical distribution of the eight sampling locations is shown in Figure 1.

**Figure 1:**
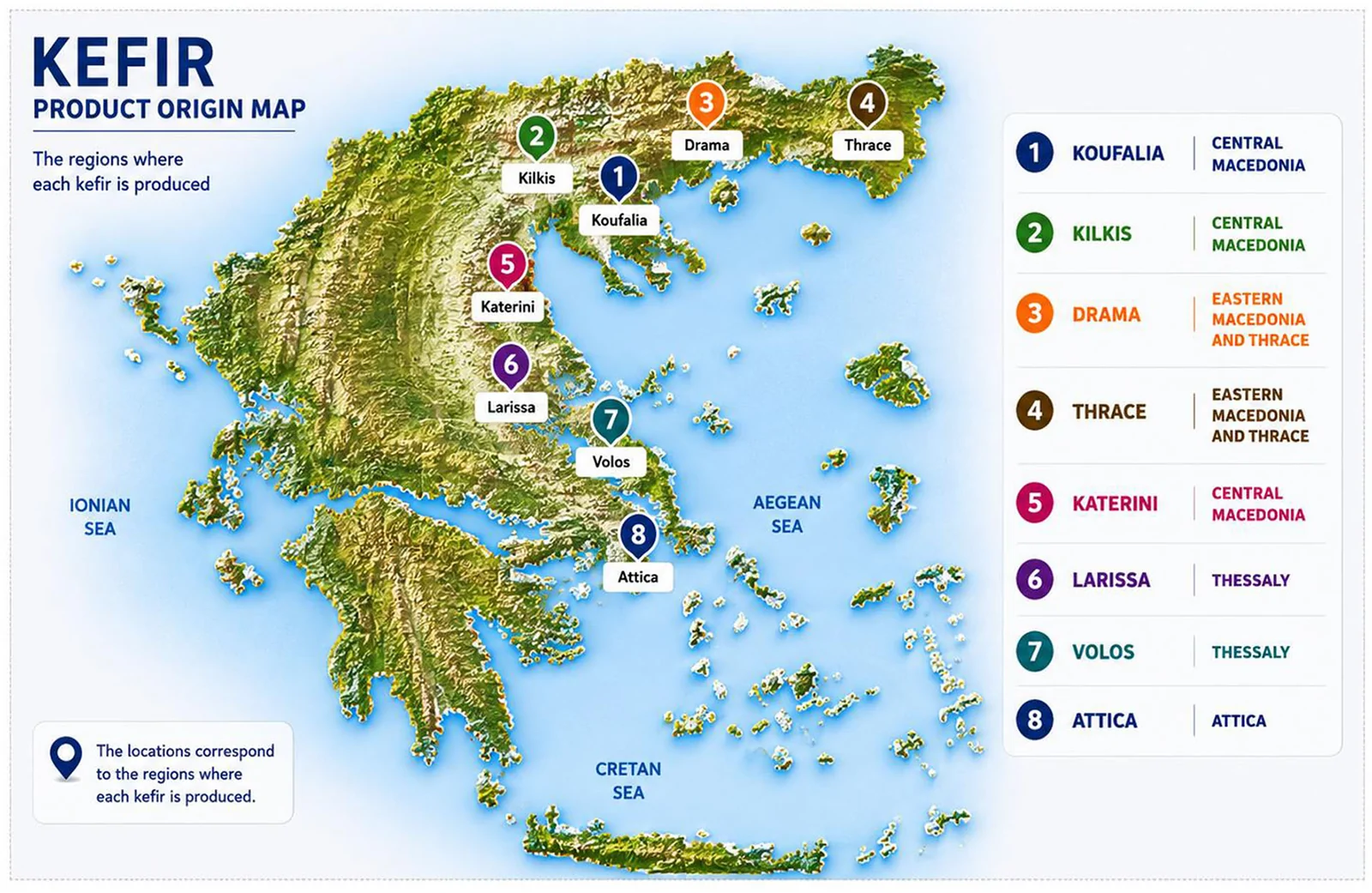
Geographical distribution of the eight sampling locations in Greece, shown according to their respective administrative regions, from which commercially produced kefir products were obtained. Central Macedonia: Koufalia, Kilkis, Katerini. Eastern Macedonia and Thrace: Drama, Thrace. Thessaly: Larissa, Volos. Attica: Attica.

### 2.2 Genomic DNA extraction

Total genomic DNA was extracted using the NucleoSpin^®^ Food Kit (Macherey-Nagel GmbH & Co. KG, Düren, Germany) according to the manufacturer's instructions. DNA concentration and purity were evaluated using a Qubit™ 4 Fluorometer (Thermo Fisher Scientific, Waltham, MA, USA). Extracted DNA samples were stored at −20 °C until further molecular analysis.

### 2.3 DNA amplification, barcoding and library preparation and 16S rRNA sequencing

Bacterial community profiling was performed through amplification of the full-length 16S rRNA gene (V1–V9 regions) using the Oxford Nanopore Technologies (ONT) Microbial Amplicon Barcoding Kit (SQK-MAB114.24). For each sample, 10 ng of genomic DNA was used as template for PCR amplification.

PCR reactions were performed in a final volume of 50 μL containing LongAmp^®^ Hot Start Taq 2 × Master Mix (New England Biolabs, Ipswich, MA, USA), primer mix supplied by ONT, and genomic DNA. Amplification conditions consisted of an initial denaturation at 95 °C for 1 min, followed by 40 cycles of 95 °C for 20 s, 55 °C for 30 s, and 65 °C for 2 min, with a final extension at 65 °C for 5 min. PCR negative controls and a positive control consisting of *Lactobacillus rhamnosus* DNA were included during amplification. No amplification was observed in the negative controls, whereas the positive control produced the expected amplification product. Control samples were used for quality-control purposes and were not included in the sequencing dataset. Extraction blanks were not included in the present experimental workflow.

PCR products were quantified using the Qubit™ 4 fluorometer and subsequently barcoded using ONT native barcode adapters. Barcoded amplicons were pooled in equimolar concentrations and purified using AMPure XP magnetic beads (Beckman Coulter, USA) at a 0.8 × bead-to-sample ratio. Sequencing libraries were prepared according to the manufacturer's protocol and loaded onto R10.4.1 flow cells. Sequencing runs were performed on the MinION platform using MinKNOW software (Oxford Nanopore Technologies, Oxford, UK).

### 2.4 Bioinformatic analysis

Raw sequencing data were basecalled using Guppy Basecaller v6.3.7 (Oxford Nanopore Technologies) with the High Accuracy (HAC) basecalling model. Demultiplexing and barcode trimming were performed during sequence processing. Only reads with a minimum quality score of *Q* ≥ 9 were retained for downstream analysis.

Sequence data were processed using the Oxford Nanopore Technologies EPI2ME wf-16S workflow (v1.6.1). Taxonomic classification was performed using the Minimap2-based classification approach against the NCBI 16S/18S ribosomal RNA reference database (ncbi_16s_18s; database release dated 6 May 2026). Reads shorter than 800 bp or longer than 2,000 bp were excluded from downstream analysis. Taxonomic assignments were retained when reads showed a minimum sequence identity of 95% to the matched reference and a minimum reference coverage of 90%; reads failing either criterion were considered unclassified.

No dedicated computational chimera-detection or chimera-removal algorithm was applied during sequence processing. Accordingly, no reads were specifically removed on the basis of chimera detection. PCR products were evaluated by agarose gel electrophoresis prior to sequencing and showed a single amplification product of the expected size without additional visible amplification products. However, electrophoretic assessment alone cannot exclude the presence of low-abundance chimeric amplicons, particularly considering the relatively high number of PCR amplification cycles used in the present study.

To evaluate sequencing-depth adequacy, three randomly selected samples were subsampled to 30,000 and 100,000 reads. Differences in taxonomic composition between the subsampled datasets were negligible (<0.1%), indicating that sequencing depth was sufficient for reliable taxonomic characterization. Therefore, 50,000 reads per sample were used for the final analyses. Alpha-rarefaction curves were generated to confirm adequate sequencing coverage and diversity saturation.

Species-level assignments, particularly among closely related *Lactococcus* taxa, were interpreted with caution because full-length 16S rRNA gene sequences may not always provide unambiguous discrimination among phylogenetically closely related species. Accordingly, species-level classifications represent the most likely taxonomic assignments generated by the bioinformatic workflow rather than definitive confirmation of species identity.

### 2.5 Statistical analysis

Relative abundance data were calculated at the phylum, genus, and species levels and expressed as percentages of the total classified reads. Beta diversity was assessed using Bray–Curtis dissimilarity based on species-level relative abundance profiles. Principal Coordinates Analysis (PCoA) was performed to visualize differences in bacterial community composition among the eight commercial product groups.

The statistical significance of differences in overall bacterial community composition among product groups was evaluated using permutational multivariate analysis of variance (PERMANOVA) with 999 permutations. Before PERMANOVA, the homogeneity of multivariate dispersion was assessed to determine whether significant differences were associated primarily with differences in community composition rather than with unequal within-group dispersion.

Alpha diversity was evaluated using the Shannon diversity index, Simpson diversity index (1-D), and observed species richness. Differences in alpha-diversity metrics among product groups were assessed using Kruskal–Wallis tests. Statistical significance was set at *p* < 0.05. To account for the compositional nature of microbiome relative-abundance data, species-level count profiles were additionally subjected to centered log-ratio (CLR) transformation prior to taxon-specific comparisons. Due to the fact that zero counts preclude logarithmic transformation, a pseudocount of 0.5 was added before CLR transformation. The transformed abundances of *Streptococcus thermophilus* and *Lactococcus cremoris* were compared among product groups, using Kruskal–Wallis tests. In the cases where significant overall differences were identified, Dunn's pairwise comparisons had been conducted with Benjamini–Hochberg false discovery rate correction.

## 3 Results

### 3.1 Overall bacterial composition of commercially produced Greek kefir

Full-length 16S rRNA gene sequencing revealed that commercially produced Greek kefir was predominantly composed of lactic acid bacteria. At the phylum level, *Bacillota* represented the dominant phylum in all analyzed samples, accounting for 92.53%−100% of the bacterial community. Low proportions of *Actinomycetota* were detected only in selected samples and were mainly represented by *Bifidobacterium animalis*.

At the genus level, the bacterial communities were primarily composed of *Streptococcus* and *Lactococcus*, indicating that the microbiota of commercially produced Greek kefir was largely associated with starter-culture organisms used in dairy fermentation. At the species level, *Streptococcus thermophilus* and *Lactococcus cremoris* constituted the dominant taxa, with mean relative abundances of 45.89% and 37.72%, respectively. Together, these two species accounted for approximately 84.7% of the total bacterial population (Figure 2).

**Figure 2:**
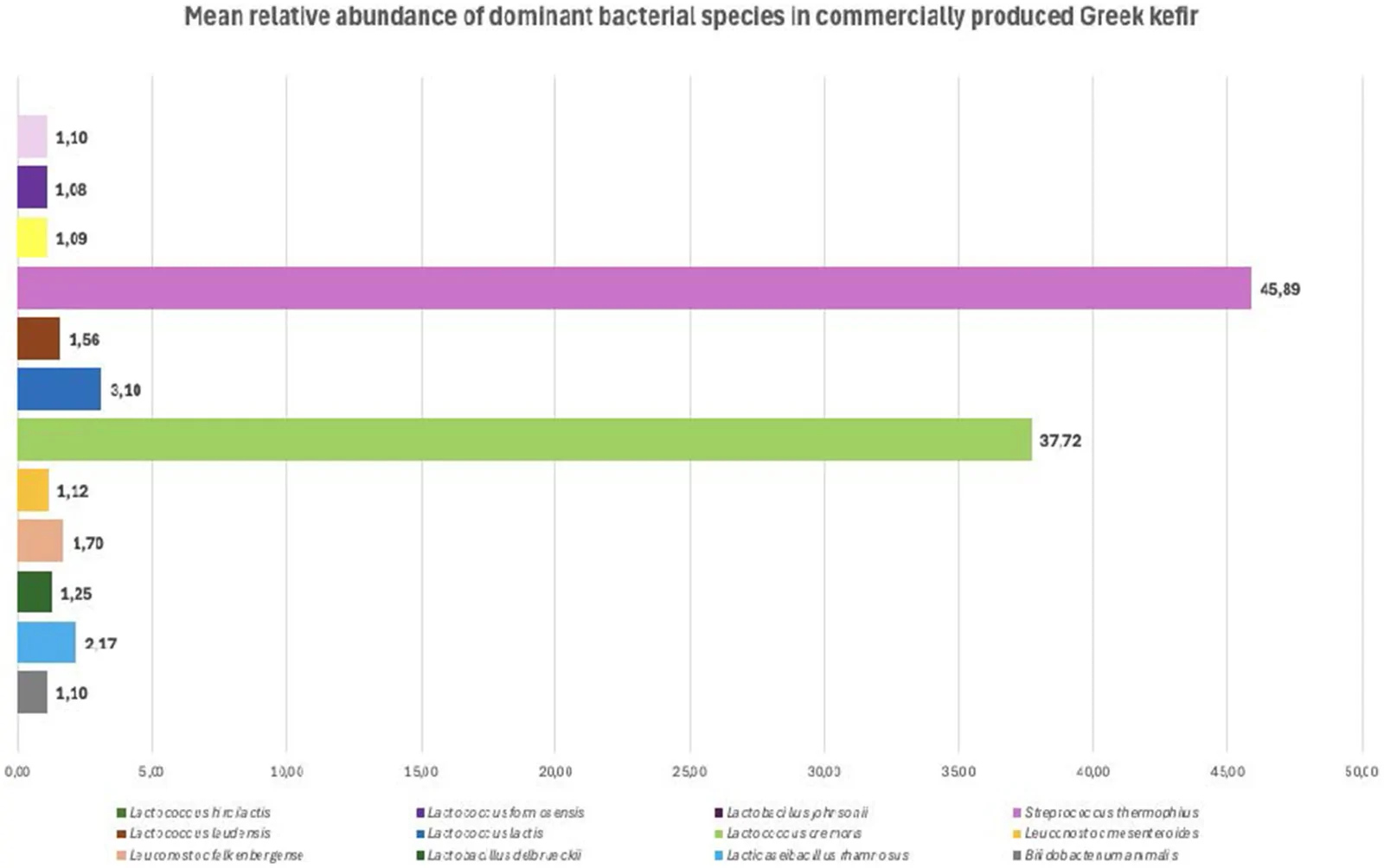
Mean relative abundance (%) of the dominant bacterial species identified in commercially produced Greek kefir products using full-length 16S rRNA gene sequencing. Taxa with lower relative abundances were grouped as “Other bacterial taxa.”

Additional species were detected at lower relative abundances, including *Lactococcus lactis* (3.10%), *Leuconostoc falkenbergense* (1.70%), *Lactococcus laudensis* (1.56%), *Lactobacillus delbrueckii* (1.25%), *Leuconostoc mesenteroides* (1.12%), *Lacticaseibacillus rhamnosus* (2.17%), *Bifidobacterium animalis* (1.10%), *Lactobacillus johnsonii* (1.09%), *Lactococcus formosensis* (1.08%), and *Lactococcus hircilactis* (1.10%). Overall, the microbial composition indicated the presence of dominant shared bacterial taxa, primarily represented by technologically important LAB (Table 1).

**Table 1:** Main bacterial taxa detected in commercially produced Greek kefir products and selected characteristics previously reported for these taxa in the literature.

Bacterial species	Main morphological and metabolic characteristics	Technological relevance in fermented dairy products	References
*Bifidobacterium animalis*	Gram-positive, anaerobic, branched rod-shaped bacterium; heterofermentative metabolism through the bifid shunt; optimum growth temperature approximately 37 °C; produces acetate and lactate.	Frequently incorporated as an adjunct culture in fermented milk and probiotic dairy products. Its technological application depends on strain-specific survival during processing, storage, and gastrointestinal transit.	O'Callaghan and van Sinderen, 2016; Hill et al., 2014
*Lacticaseibacillus rhamnosus*	Gram-positive, rod-shaped, facultatively heterofermentative bacterium; mesophilic growth, generally between 30 °C and 37 °C; several strains exhibit tolerance to acidic conditions and bile salts.	Widely used as an adjunct culture in fermented milk, yogurt, cheese, and kefir-type products. Certain strains may produce organic acids and antimicrobial compounds.	Yang et al., 2026
*Lactobacillus delbrueckii*	Gram-positive, rod-shaped, homofermentative and thermophilic bacterium; optimum growth generally between 42 °C and 45 °C; characterized by rapid lactose fermentation and proteolytic activity.	Important dairy starter bacterium contributing to acidification, flavor development, acetaldehyde production, and texture. Frequently used in association with *Streptococcus thermophilus*.	Nami et al., 2026
*Lactobacillus johnsonii*	Gram-positive, rod-shaped and homofermentative bacterium; grows optimally at approximately 37 °C−42 °C; produces lactic acid and, in some strains, antimicrobial compounds.	Used mainly as an adjunct culture in probiotic and fermented dairy products. Its technological and functional properties are strain dependent.	Arzola-Martínez et al., 2024
*Lactococcus cremoris*	Gram-positive, coccoid, homofermentative and mesophilic bacterium; optimum growth generally between 25 °C and 30 °C; efficiently converts lactose into lactic acid.	Important dairy starter culture involved in acidification, proteolysis, flavor formation, and texture development. Some strains produce exopolysaccharides or bacteriocins.	Manno et al., 2018
*Lactococcus formosensis*	Gram-positive, coccoid, homofermentative and mesophilic bacterium initially isolated from fermented food.	Its technological properties in dairy fermentation remain insufficiently characterized. Potential use as a starter or adjunct culture requires further investigation.	Chen et al., 2014
*Lactococcus hircilactis*	Gram-positive, coccoid, homofermentative and mesophilic bacterium originally isolated from milk.	May contribute to milk acidification and aroma development. Its suitability as a dairy starter or adjunct culture is still under investigation.	Meucci et al., 2015; Tidona et al., 2018
*Lactococcus lactis*	Gram-positive, coccoid, homofermentative and mesophilic bacterium; optimum growth generally between 25 °C and 30 °C; rapidly produces lactic acid from lactose.	One of the most important dairy starter cultures, contributing to acidification, proteolysis, flavor development, and product preservation. Several strains produce bacteriocins, including nisin.	Song et al., 2017
*Lactococcus laudensis*	Gram-positive, coccoid, homofermentative and mesophilic bacterium originally isolated from milk.	May contribute to acidification and aroma formation in dairy products. Its technological properties require further characterization.	Meucci et al., 2015; Tidona et al., 2018
*Leuconostoc falkenbergense*	Gram-positive, coccoid, obligately heterofermentative and mesophilic bacterium; produces lactic acid, carbon dioxide, ethanol or acetate, and extracellular polysaccharides.	May contribute to aroma, gas formation, and texture development in fermented foods. Its industrial dairy applications remain under investigation.	Wu and Gu, 2021; Llamas-Arriba et al., 2021
*Leuconostoc mesenteroides*	Gram-positive, coccoid, obligately heterofermentative and mesophilic bacterium; produces lactic acid, carbon dioxide, diacetyl, acetoin, and extracellular polysaccharides.	Used in several dairy fermentations because of its contribution to aroma, flavor, texture, and gas formation. Some strains may also produce antimicrobial compounds.	de Paula et al., 2015
*Streptococcus thermophilus*	Gram-positive, coccoid, homofermentative and thermophilic bacterium; optimum growth generally between 40 °C and 45 °C; rapidly ferments lactose and may produce exopolysaccharides.	Major dairy starter bacterium contributing to acidification, flavor, texture, and product stability. Commonly used in association with *Lactobacillus delbrueckii*.	Iyer et al., 2010

The technological and potential functional characteristics presented in this table are literature-based and do not represent experimentally confirmed properties of the bacterial populations detected in the present study.

### 3.2 Variation in bacterial communities among product groups

Despite the presence of dominant shared bacterial taxa, substantial variation was observed among the analyzed commercial kefir product groups. Bacterial profiles are presented as mean relative abundances calculated from four independent kefir samples per sampling group.

Samples from Koufalia were strongly dominated by *Lactococcus cremoris*, which represented 88.59% of the total bacterial community, whereas *Streptococcus thermophilus* accounted for only 4.34%. At the genus level, *Lactococcus* represented approximately 92.05% of the community.

In contrast, samples from Attica, Thrace, and Kilkis were characterized by the predominance of *S. thermophilus*. The relative abundance of this species was 74.33% in Attica, 66.67% in Thrace, and 64.58% in Kilkis. The Thrace product group exhibited a particularly simple bacterial profile, consisting almost exclusively of *S. thermophilus* and *L. cremoris*.

Kefir samples from Drama, Larissa, and Volos displayed intermediate profiles characterized by the co-dominance of *S. thermophilus* and *L. cremoris*. In Drama, *L. cremoris* was predominant at 51.25%, followed by *S. thermophilus* at 29.95%, whereas Larissa and Volos showed more balanced distributions of the two dominant taxa.

Samples from Katerini showed a compositionally heterogeneous bacterial profile, that is, characterized by a relatively broad distribution of bacterial taxa and comparatively low dominance of the two core species. No individual species exceeded 40% relative abundance. The dominant species was *S. thermophilus* (36.28%), followed by *Leuconostoc falkenbergense* (10.25%), *Lactococcus lactis* (9.27%), *Leuconostoc mesenteroides* (9.15%), *L. cremoris* (8.29%), *Bifidobacterium animalis* (7.47%), *Lacticaseibacillus rhamnosus* (5.80%), and *Lactobacillus delbrueckii* (3.04%). Katerini was also the only product group with a substantial contribution of Actinomycetota, mainly due to the presence of *B. animalis*.

Also, alpha-diversity analysis showed significant overall differences among product groups for Shannon diversity [*H*_(7)_ = 24.625, *p* < 0.001], Simpson diversity [*H*_(7)_ = 18.688, *p* = 0.009] and observed richness [*H*_(7)_ = 30.827, *p* < 0.001]. Katerini showed comparatively high Shannon and Simpson diversity. However, it did not present the highest values for any of the evaluated alpha-diversity metrics. Drama showed the highest mean Shannon and Simpson diversity and Volos presented the highest observed richness.

### 3.3 Beta diversity and clustering among product groups

Bray–Curtis PCoA demonstrated clear clustering of kefir samples according to their commercial product group (Figure 3). The first three principal coordinates explained 84.84% of the total variation in bacterial community composition, with PC1 explaining 64.25%, PC2 explaining 15.52%, and PC3 explaining 5.07%.

**Figure 3:**
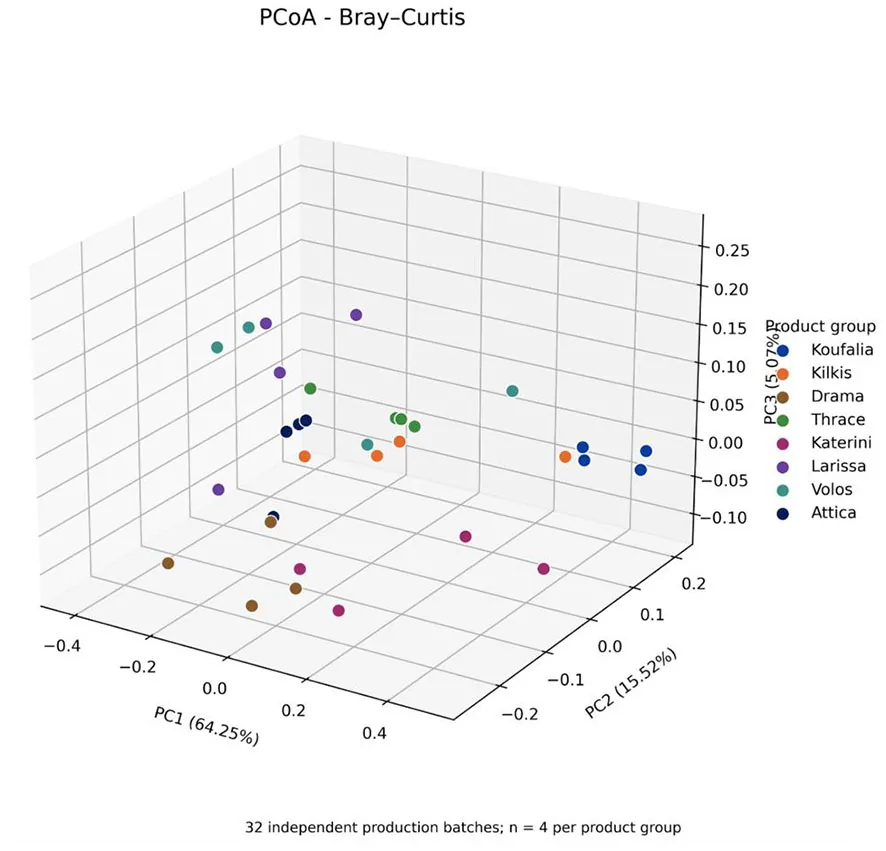
Three-dimensional principal coordinates analysis (PCoA) based on Bray–Curtis dissimilarity of bacterial community composition in 32 independent commercially produced Greek kefir samples. Each point represents one independent production batch, with four independent batches included for each of the eight commercial product groups (*n* = 4 per group; total *n* = 32). Analytical duplicates were not included as separate observations. The first three principal coordinates explained 84.84% of the total variation in community composition (PC1: 64.25%, PC2: 15.52%, PC3: 5.07%).

The separation along PC1 was consistent with marked differences in the relative abundances of the dominant LAB species, particularly *Lactococcus cremoris* and *Streptococcus thermophilus*. Koufalia formed a clearly distinct cluster along PC1, consistent with its marked predominance of *Lactococcus cremoris* and low abundance of *Streptococcus thermophilus*.

PC2 further separated Katerini from the remaining regions. This separation was consistent with the distinct composition of Katerini samples, which included substantial proportions of *Leuconostoc* spp., *Lacticaseibacillus rhamnosus, Bifidobacterium animalis, Lactococcus lactis*, and *Lactobacillus delbrueckii*.

PC3 accounted for an additional 5.07% of the total variation and provided limited additional separation among product-group clusters.

The compact clustering of samples within most commercial product groups suggested relatively high within-group similarity, whereas the separation among clusters indicated differences in bacterial community composition among commercial kefir products.

PERMANOVA based on Bray–Curtis dissimilarities presented statistically significant differentiation in bacterial community composition among product groups (pseudo-*F* = 5.630, *R*^2^ = 0.622, permutation *p* = 0.001; 999 permutations). The PERMANOVA partitioning yielded seven degrees of freedom and a sum of squares of 2.4357 for product group, and 24 residual degrees of freedom. There was also a residual sum of squares of 1.4833 (total SS = 3.9190). The multivariate dispersion test did not detect statistically significant differences in dispersion among product groups [*F*_(7, 24)_ = 2.939, permutation *p* = 0.099]. This shows that no significant heterogeneity of dispersion was detected.

### 3.4 Statistical comparison of the dominant bacterial species

Because microbiome relative-abundance data are compositional, the two dominant bacterial species were additionally evaluated after centered log-ratio (CLR) transformation. CLR-based Kruskal–Wallis analysis showed statistically significant overall differences among product groups for both *Streptococcus thermophilus* and *Lactococcus cremoris*.

Specifically, for *Streptococcus thermophilus*, significant variation among product groups was detected [*H*_(7)_ = 22.483, *p* = 0.002]. Dunn's *post-hoc* comparisons with Benjamini–Hochberg correction presented statistically significant differences between Koufalia and Thrace (adjusted *p* = 0.0085), Koufalia and Attica (adjusted *p* = 0.0085), Thrace and Katerini (adjusted *p* = 0.0159), and Attica and Katerini (adjusted *p* = 0.0159).

For *Lactococcus cremoris*, statistically significant overall differences were also observed [*H*_(7)_ = 17.591, *p* = 0.014]. Significant pairwise differences were detected between Koufalia and Volos (adjusted *p* = 0.0329), Koufalia and Larissa (adjusted *p* = 0.0329), Koufalia and Drama (adjusted *p* = 0.0329), and Koufalia and Attica (adjusted *p* = 0.0194). Therefore, the CLR-based results supported the presence of group-related differences in the two dominant taxa, while accounting for the compositional structure of the microbiome data.

The sample-level bacterial composition of all 32 kefir samples is presented in Figure 4. Visualization of the four independent production batches within each commercial product group illustrates both within-group variation and the marked compositional differences observed among product groups.

**Figure 4:**
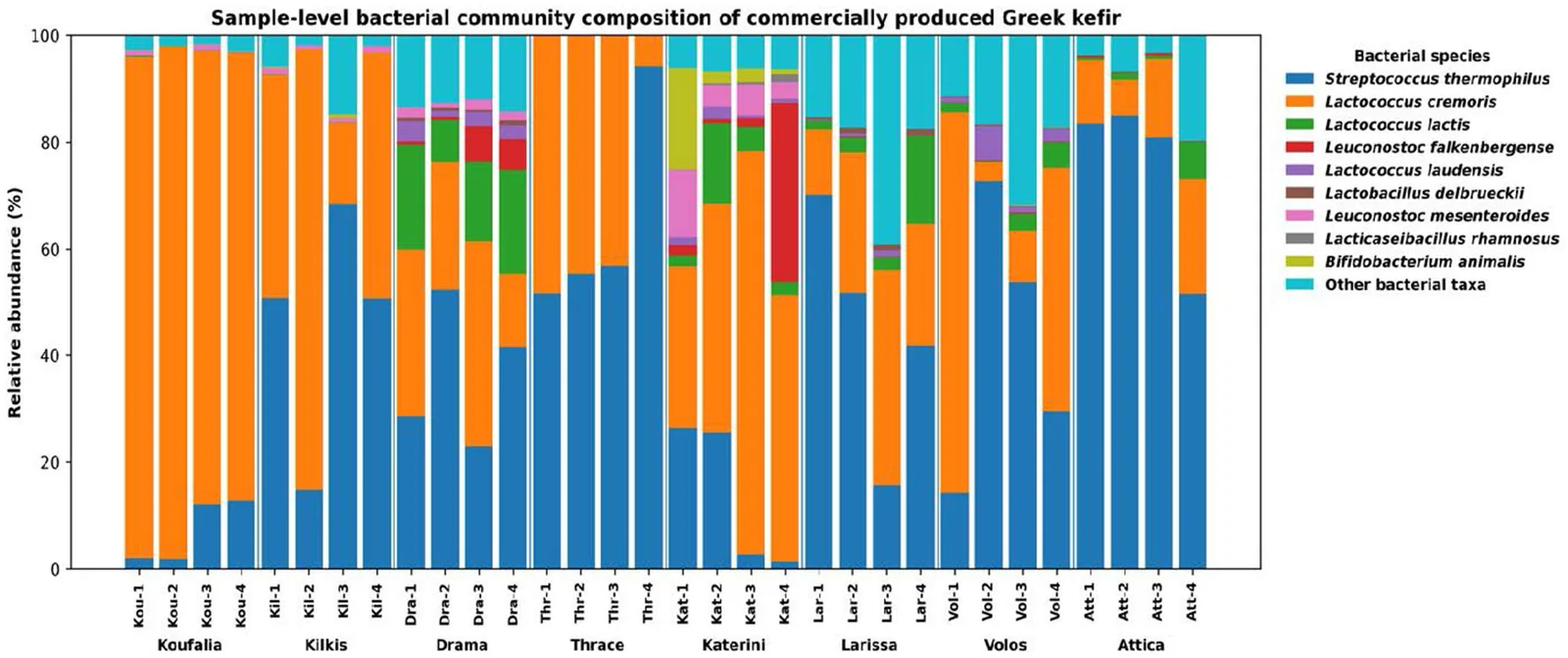
Sample-level relative abundance (%) of the dominant bacterial species identified in 32 commercially produced Greek kefir samples using full-length 16S rRNA gene sequencing. Four independent production batches are shown for each commercial product group. Taxa not individually displayed were grouped as “Other bacterial taxa.”

### 3.5 Product group-specific bacterial profiles

#### 3.5.1 Koufalia (Central Macedonia)

Kefir samples from Koufalia displayed a bacterial composition strongly dominated by *Lactococcus cremoris* (88.59%), while *Streptococcus thermophilus* represented only 4.34%. All remaining bacterial species occurred at relative abundances below 1.5%, including *Bifidobacterium animalis, Leuconostoc mesenteroides, Lactobacillus johnsonii, Lactococcus formosensis*, and *Lactococcus hircilactis*.

#### 3.5.2 Volos (Thessaly)

Kefir samples from Volos exhibited a more heterogeneous bacterial composition. The dominant species were *Streptococcus thermophilus* (43.32%) and *Lactococcus cremoris* (36.42%), followed by *Lactococcus lactis* (7.31%) and *Lactococcus laudensis* (4.62%). Minor populations included *Bifidobacterium animalis* (2.05%), *Lactobacillus delbrueckii* (2.19%), *Lactococcus formosensis* (2.06%), and *Lactococcus hircilactis* (2.04%).

#### 3.5.3 Larissa (Thessaly)

Kefir samples from Larissa were characterized by the predominance of *Streptococcus thermophilus* (47.62%) and *Lactococcus cremoris* (35.09%). Additional bacterial species included *Lactococcus laudensis* (7.58%), *Lactococcus lactis* (2.80%), *Lactobacillus delbrueckii* (2.73%), *Leuconostoc falkenbergense* (2.13%), and *Lactococcus hircilactis* (2.04%).

#### 3.5.4 Drama (Thrace)

The bacterial community of the Drama samples was dominated by *Lactococcus cremoris* (51.25%), followed by *Streptococcus thermophilus* (29.95%) and *Lactococcus lactis* (8.55%). Lower relative abundances were observed for *Leuconostoc falkenbergense* (2.64%), *Lactococcus laudensis* (1.91%), *Lactobacillus delbrueckii* (1.79%), *Leuconostoc mesenteroides* (1.55%), *Lactococcus formosensis* (1.28%), and *Lactococcus hircilactis* (1.07%).

#### 3.5.5 Kilkis (Central Macedonia)

The Kilkis kefir samples were strongly dominated by *Streptococcus thermophilus*, which accounted for 64.58% of the total bacterial population. The second most abundant species was *Lactococcus cremoris* (27.91%), while all remaining taxa were present at relative abundances below 2%, including *Leuconostoc mesenteroides, Bifidobacterium animalis, Lactobacillus johnsonii, Lactococcus formosensis*, and *Lactococcus hircilactis*.

#### 3.5.6 Thrace

The Thrace samples exhibited the least compositionally heterogeneous bacterial profile among the analyzed product groups, consisting almost exclusively of two bacterial species: *Streptococcus thermophilus* (66.67%) and *Lactococcus cremoris* (33.33%). No additional bacterial taxa were detected above the applied analysis threshold.

#### 3.5.7 Attica

Kefir samples from Attica showed the highest relative abundance of *Streptococcus thermophilus* among all analyzed product groups, reaching 74.33%. The remaining bacterial population mainly consisted of *Lactococcus cremoris* (20.84%), while *Lactococcus lactis, Lactococcus formosensis*, and *Lactococcus hircilactis* were detected at low relative abundances.

#### 3.5.8 Katerini (Central Macedonia)

Among all analyzed product groups, Katerini exhibited the most compositionally heterogeneous bacterial profile and the lowest dominance of the two core taxa. No individual bacterial species exceeded 40% relative abundance. The bacterial community was dominated by *Streptococcus thermophilus* (36.28%), followed by *Leuconostoc falkenbergense* (10.25%), *Lactococcus lactis* (9.27%), *Leuconostoc mesenteroides* (9.15%), *Lactococcus cremoris* (8.29%), *Bifidobacterium animalis* (7.47%), *Lacticaseibacillus rhamnosus* (5.80%), and *Lactobacillus delbrueckii* (3.04%). This product group was the only one showing comparatively high relative abundances of both *Lacticaseibacillus rhamnosus* and *Leuconostoc* species, indicating a more complex bacterial fermentation profile.

## 4 Discussion

A key strength of the present study is the application of full-length 16S rRNA gene sequencing, which enabled detailed characterization of the dominant bacterial taxa in commercially produced Greek kefir products. The results demonstrated that *Streptococcus thermophilus* and *Lactococcus cremoris* were the dominant shared taxa across the analyzed products. Although these taxa predominated overall, their relative abundances varied markedly among the commercial product groups, indicating substantial differences in the bacterial composition of the analyzed kefir products.

The predominance of *S. thermophilus* and *L. cremoris* is consistent with the widespread use of technologically important lactic acid bacteria in industrial dairy fermentations. *S. thermophilus* is commonly used as a starter culture because of its rapid acidification capacity, contribution to flavor development, and role in product stability. Similarly, *L. cremoris* is an important dairy-associated species that contributes to acid production, texture formation, and flavor development during fermentation. Its association with exopolysaccharide production and bioactive peptide formation may also influence the sensory and technological characteristics of fermented dairy products (Ahmed et al., 2013; Guzel-Seydim et al., 2011).

The bacterial composition identified in the present study differed from that previously reported for traditional kefir grains. Kalamaki and Angelidis (2020) reported that Greek kefir grains were predominantly characterized by *Lactobacillus kefiranofaciens, Lactobacillus kefiri*, and *Lactobacillus parakefiri*. In contrast, the commercially produced kefir products analyzed in the present study were largely dominated by *S. thermophilus* and *L. cremoris*. These differences may reflect the use of defined starter cultures, controlled industrial fermentation conditions, and product standardization practices. In traditional kefir grains, repeated propagation of a complex microbial consortium may support the maintenance of a broader range of interacting bacterial populations, whereas industrial manufacture commonly relies on selected starter cultures designed to provide reproducible acidification, sensory characteristics, and processing performance. Such standardization may favor a smaller number of technologically desirable taxa and thereby reduce part of the compositional complexity typically associated with traditional kefir ecosystems. At the same time, the marked differences observed among the commercial product groups in the present study indicate that industrial production does not necessarily result in complete microbiological uniformity. Differences in starter formulation, milk substrate, fermentation parameters, adjunct cultures, and product formulation may still generate distinct bacterial profiles among commercial kefir products.

Similar differences between traditional kefir grains, homemade kefir, and industrial kefir beverages have also been reported previously (Kazou et al., 2021; Beshkova et al., 2002; Sarkar, 2008).

Also, the taxon-specific comparisons were further examined using CLR-transformed data to account for the compositional nature of microbiome profiles. Significant overall differences existed for both *S. thermophilus* and *L. cremoris* after CLR transformation. This shows that the observed group-related patterns were not solely an artifact of direct percentage-based comparisons. Nevertheless, taxon-specific relative abundances are still intrinsically dependent on the composition of the overall microbial community. Therefore, these results should be assessed together with the multivariate community-level analyses and not in isolation.

The analyzed commercial product groups displayed distinct bacterial profiles. Kefir products from Koufalia and Drama were characterized by high relative abundances of *L. cremoris*, whereas products from Attica, Kilkis, and Thrace showed higher relative abundances of *S. thermophilus*. These differences should not be interpreted as an effect of geographical location alone. Instead, the sampling groups may reflect variation in starter culture formulations, milk source, fermentation conditions, industrial processing, storage, and manufacturer-specific practices. Previous studies have shown that milk type and starter culture composition may influence the physicochemical, rheological, sensory, and microbial characteristics of kefir products (Wszolek et al., 2001; Tratnik et al., 2006; Öner et al., 2010; de Sainz et al., 2020; Barukčić et al., 2017). Katerini exhibited compositionally distinct and relatively heterogeneous bacterial profile among the analyzed commercial product groups. In addition to *S. thermophilus* and *L. cremoris*, this group showed comparatively higher relative abundances of *Leuconostoc falkenbergense, Leuconostoc mesenteroides, Lactococcus lactis, Lacticaseibacillus rhamnosus, Lactobacillus delbrueckii*, and *Bifidobacterium animalis*. This broader taxonomic distribution may indicate the use of a more complex or multi-strain starter formulation, the inclusion of adjunct cultures, or differences in milk substrate and fermentation conditions compared with the other commercial products. For example, variation in fermentation temperature, fermentation duration, inoculum composition, or post-fermentation storage conditions could differentially favor mesophilic and thermophilic LAB and contribute to the lower dominance of *S. thermophilus* and *L. cremoris* observed in this product group. The detection of *B. animalis* and comparatively higher abundances of *L. rhamnosus* may also be compatible with the use of adjunct cultures in the product formulation. However, because detailed manufacturing and formulation data were not available for all products, these explanations remain hypotheses and cannot be confirmed by the present dataset. Taxa belonging to the genera *Leuconostoc, Lacticaseibacillus*, and *Bifidobacterium* identified in this group include species that have previously been associated with technological or probiotic-related properties in fermented dairy systems (Ahmed et al., 2013; Azizi et al., 2021). However, the present DNA-based sequencing approach does not establish the viability, strain identity, or functional activity of these organisms in the analyzed products.

However, the alpha-diversity analysis did not identify Katerini as the product group with the highest bacterial diversity. Rather, Katerini showed comparatively high Shannon and Simpson diversity. At the same time, Drama presented the highest mean values for these indices. Then, Volos showed the highest observed richness. Thus, the heterogeneity that was observed in the Katerini compositional profile should not be interpreted as evidence that this group had the highest alpha diversity.

The observed variation among the analyzed commercial kefir products is consistent with recent retail-based studies. Maughan et al. (2025) reported considerable variation in bacterial and fungal composition among retail kefir products available in Ireland, whereas Metras et al. (2021) demonstrated that the microbial composition and density of commercial kefir products may differ and may not always correspond fully with the microoganisms declared on product labels. These findings emphasize that commercially available kefir products should not be considered microbiologically uniform, even when marketed under the same general product category.

Previous studies have suggested that differences in kefir microbial consortia may be associated with differences in fermentation-derived metabolites and biological effects; however, such functional properties cannot be inferred from taxonomic profiling alone. Bourrie et al. (2021) demonstrated that kefir products with different microbial compositions produced distinct effects on cholesterol and lipid metabolism in a mouse model of diet-induced obesity. In human subjects, kefir consumption has also been associated with changes in selected gut microbiota-related outcomes (Bellikci-Koyu et al., 2019).

Recent metagenomic studies of fermented ecosystems further demonstrate that changes in microbial community composition may be accompanied by substantial shifts in functional gene content and metabolic pathways. For example, whole-genome metagenomic analysis of natural food-waste fermentation revealed progressive microbial succession toward a lactic acid bacteria-dominated community together with changes in gene-family richness and metabolic functions related to carbohydrate metabolism, fermentation, transport, and stress response (Parab et al., 2026). Similar metagenomic approaches in fermented food systems have linked microbial succession with stage-specific metabolic pathways and functional potential. These findings emphasize that taxonomic profiles obtained by 16S rRNA gene sequencing cannot alone be used to infer the functional activity of the detected microbial communities and support the application of metagenomic approaches in future studies of commercial kefir.

An important limitation of the present study is that full-length 16S rRNA gene sequencing is DNA-based and therefore cannot distinguish DNA originating from viable bacterial cells from DNA derived from dead cells or extracellular cell-free DNA. Consequently, taxonomic detection should not be interpreted as direct evidence of microbial viability, metabolic activity, probiotic functionality, or health-promoting effects. In addition, 16S rRNA gene sequencing provides taxonomic information but does not directly characterize functional gene content or metabolite production. Therefore, any functional interpretation of the detected taxa should be considered hypothesis-generating and based on previously reported characteristics of related strains or species rather than on experimentally demonstrated functions in the present samples. Future studies combining viability-PCR or culture-based enumeration with metagenomic, metatranscriptomic, and metabolomic approaches would allow discrimination between viable and non-viable microbial signals and provide a more direct assessment of the functional potential of commercial kefir microbiota.

Overall, the present findings provide a comparative overview of the bacterial microbiota of commercially produced Greek kefir products and demonstrate that, despite the presence of dominant shared lactic acid bacteria, marked variation exists among the analyzed commercial product groups. The observed variation among commercial product groups may be associated with differences in starter culture composition, milk source, product formulation, storage conditions, and manufacturer-specific practices. However, these factors were not experimentally evaluated as independent variables in the present study, and their relative contribution to the observed bacterial-community differences cannot be determined from the current dataset.

## 5 Conclusions

The present study provides a comparative characterization of the bacterial communities associated with commercially produced Greek kefir products collected from different regions of Greece using full-length 16S rRNA gene sequencing. Across all analyzed samples, *Streptococcus thermophilus* and *Lactococcus cremoris* represented the dominant shared bacterial taxa. Despite the predominance of these two species, marked variation was observed among the commercial product groups, with Katerini showing a compositionally distinct and relatively heterogeneous bacterial profile. Alpha-diversity analysis further demonstrated significant differences among product groups; however, Katerini did not exhibit the highest diversity across the evaluated indices.

The application of Oxford Nanopore full-length 16S rRNA gene sequencing enabled detailed taxonomic characterization of the dominant bacterial communities and highlighted substantial variation among the analyzed commercial kefir products. However, the present DNA-based approach does not provide information on microbial viability, metabolic activity, functional gene expression, or probiotic activity. Accordingly, the functional significance of the detected bacterial taxa should not be inferred directly from their taxonomic identification.

The observed differences among commercial product groups may be associated with several product- and manufacturer-related factors, including starter culture composition, milk source, formulation, processing conditions, and storage. However, the relative contribution of these factors cannot be determined from the present dataset alone. Future studies integrating detailed product metadata, microbial viability assessment, culture-based approaches, metagenomic and metabolomic analyses, and physicochemical characterization will be valuable for clarifying the factors that shape commercial kefir microbiota and their relationship with technological, sensory, and potential functional properties.

## Data Availability

The original contributions presented in the study are available at this link: https://doi.org/10.5281/zenodo.23035279.
