# Supplementary material for: Marked variation in the bacterial microbiota of commercially produced Greek kefir products revealed by full-length 16S rRNA gene sequencing
Source: Front Microbiol. 2026 Oct 7;17:1951948. doi: 10.3389/fmicb.2026.1951948 (PMC13683812; doi:10.3389/fmicb.2026.1951948)
Supplement: Supplementary file 1 [file Table_1.pdf]

Supplementary Material

Marked variation in the bacterial microbiota of commercially produced Greek kefir products revealed by full-length 16S rRNA gene sequencing

Supplementary Table S1. Product and label information for the commercially produced Greek kefir products included in the study.

Sampling group	Number of independent batches	Milk source	Declared starter culture / microorganisms	Storage conditions	Product type / formulation	Other label information
Koufalia	4	Semi-skimmed cow milk	Kefir culture	+6 °C	Cow milk with kefir culture	Consume within 3 days after opening; serve chilled
Kilkis	4	Pasteurised goat milk	Kefir culture (Bifidobacterium lactis)	+2 °C to +6 °C	Organic farming product; fermented kefir milk	Consume within 4 days after opening
Drama	4	Semi-skimmed goat milk	Kefir culture	+2 °C to +6 °C	Organic farming product	Shake before use; consume within 3 days after opening
Thrace	4	Semi-skimmed, pasteurised cow milk	Kefir culture	≤ +7 °C	Cow milk with kefir culture	Consume within 3 days after opening
Katerini	4	Semi-skimmed, pasteurised cow milk	Kefir culture	≤ +6 °C	Cow milk with kefir culture	Consume within 3 days after opening
Larissa	4	Semi-skimmed, pasteurised cow milk	Kefir culture	+2 °C to +4 °C	Cow milk with kefir culture; strawberry-flavoured product	Consume within 3 days after opening
Volos	4	Semi-skimmed cow milk	Kefir culture	+2 °C to +4 °C	Cow milk with kefir culture	Shake before use; consume within 4 days after opening
Attica	4	Semi-skimmed, pasteurised cow milk	Kefir culture	≤ +6 °C	Cow milk with kefir culture	Shake before use

Note. Information was transcribed from commercially available product labels. Manufacturer identities were anonymized.
